# *Francisella tularensis,* Portugal

**DOI:** 10.3201/eid1304.060714

**Published:** 2007-04

**Authors:** Isabel Lopes de Carvalho, Raquel Escudero, Cristina García-Amil, Helena Falcão, Pedro Anda, Maria Sofia Núncio

**Affiliations:** * Instituto Nacional de Saúde Dr. Ricardo Jorge, Águas de Moura, Portugal; † Instituto de Salud Carlos III, Majadahonda, Spain; ‡ Organização dos Produtores Pecuários do Mogadouro, Mogadouro, Portugal

**Keywords:** Francisella, tularemia, Portugal, letter

**To the Editor:** Tularemia is a zoonosis caused by *Francisella tularensis*. Recently, tularemia has emerged in new locations, populations, and settings (*1*). After an outbreak in Spain in 1997 (*2*), it was expected that the disease would spread toward Portugal, a country with an extended area that borders the affected areas.

To evaluate the situation, a surveillance project, including a seroepidemiologic study in human populations and detection of the nucleic acid of *F. tularensis* in biologic samples, was initiated. The district of Bragança, in northern Portugal, was selected as study area for its vicinity with tularemia-endemic areas of Spain and because *Dermacentor reticulatus* and *Ixodes ricinus* are well documented there (*3*).

Biologic samples were collected from 74 persons living in the study region whose activities represented an increased risk for contact with ticks and wild mammals. Serum samples were available from 48 and were analyzed with the microagglutination test (*4*). From the other 26 persons, blood samples were collected and frozen. Because of hemolization these samples were only subjected to PCR. DNA was extracted by using the QIAamp blood kit (QIAGEN GmbH, Hilden, Germany).

A total of 110 ticks were collected from vegetation by using the flagging method (n = 5) or from vertebrate hosts (n = 105) and were identified at the species level and processed individually (*5*). Of these ticks, 79 were *D. reticulates,* 1 *I. ricinus,* 15 *D. marginatus,* 11 *Rhipicephalus sanguineus,* and 4 *Hyalomma marginatum*.

A fragment of the gene encoding the 17-kDa lipoprotein (Tul4) of *F. tularensis* was amplified, as described previously (*6*). Resulting products were subjected to electrophoresis on 0.8% low-melt agarose gels (Roche Diagnostics GmbH, Mannheim, Germany), and the bands were purified by using the QIAquick gel extraction kit (QIAGEN GmbH) and sequenced with the BigDye Terminator Cycle Sequencing kit (Applied Biosystems, Foster City, CA, USA) on an ABI 377 DNA sequencer. The sequences were aligned with other sequences from databases by using ClustalX (*7*). Pairwise distance matrices were determined by the Kimura 2-parameter method, with MEGA3 software. Phylogenetic trees were constructed with the neighbor-joining algorithm, by using bootstrap analysis with 500 replications for evaluation of the matrices’ topology. Also, 1 region with short sequence tandem repeats (SSTR9) of *F. tularensis* was amplified as described previously (*8*). Resulting products were subjected to electrophoresis on a 3% MS-4 agarose gel (Pronadisa, Madrid, Spain).

The 48 samples studied by serology were negative. From the 26 human samples available for PCR, 1 was positive in the amplification of Tul4, which represented a prevalence rate of 3.8% of the samples studied. This result was confirmed by repeating both the DNA extraction and the PCR 3×. The amplification of SSTR9 in this case was negative. The difference between the results of the PCR methods targeting Tul4 and SSTR9 in the human sample is not surprising, since Tul4 PCR has higher sensitivity than that of SSTR9, which is a method not optimal for direct use in clinical samples (*8*,*9*). This positive result was for a 43-year-old man, a hunter who had frequent contact with lagomorphs. At the time of the collection, he was asymptomatic, but a history of a recent febrile illness was reported. He also stated that he had no recent occupational or recreational exposure in Spain. For the ticks, 1 female *D. reticulatus*, collected from a sheep, was positive in the amplification of Tul4 and SSTR9, with a prevalence rate of 1.3% for *D. reticulatus* and 0.9% considering the total of ticks studied.

Sequence analyses of the 2 positive samples obtained (PoHuF1 and PoTiF1 for human and tick, respectively) showed a homology of 100% with *F. tularensis*. A phylogenetic analysis based on the same sequence also grouped the samples from tick and human with *F. tularensis* subsp. *holarctica* live vaccine strain (Figure).

**Figure:**
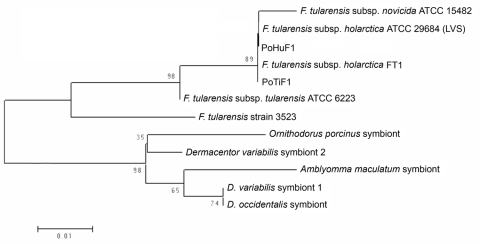
Neighbor-joining phylogenetic tree based on partial sequences of the gene coding the 17-kDa lipoprotein of *Francisella tularensis*.

This study enabled the first report of *F. tularensis* DNA detection in humans and ticks from Portugal. When studying asymptomatic persons, the likelihood of obtaining a PCR-positive result in a sample from a bacteremic patient would be expected to be much lower than finding a seropositive result. However, we did obtain a PCR-positive result from a blood sample of a person, who, as mentioned before, had a previous tularemia-compatible febrile illness. Indeed, in a previous study of 203 blood donors performed in the same area during 2001–2002, a seroprevalence rate of 8.9% in asymptomatic persons was found (*10*).

The low prevalence rates we detected contribute to the assumption that this disease should have a low incidence in Portugal, as it does in Spain. The results of this study represent the first direct evidence of *F. tularensis* in Portugal. Further studies to confirm the occurrence of human cases are needed.

GenBank accession numbers for the sequences generated in this study are DQ459299 for PoHuF1, DQ459300 for PotiF1, and DQ665890 for FT1.
